# Muscular toxicity of colchicine combined with statins: a real-world study based on the FDA adverse event reporting system database from 2004–2023

**DOI:** 10.3389/fphar.2024.1418498

**Published:** 2024-07-26

**Authors:** Ying Liu, Chunyan Wei, Yanling Yuan, Dan Zou, Bin Wu

**Affiliations:** Department of Pharmacy, West China Hospital, Sichuan University, Chengdu, China

**Keywords:** colchicine, statin, drug-related myopathy, FAERS database, drug combination

## Abstract

**Background:**

Through an analysis of the Food and Drug Administration Adverse Event Reporting System (FAERS), we explored the signal strength of adverse reactions (ADRs) related to myopathy caused by the combination of colchicine and statins and gained insight into the characteristics of these myopathy related ADRs.

**Methods:**

We extracted data from the FAERS database about ADRs in individuals with myopathy resulting from the combination of colchicine and statins. The analysis was conducted for the period spanning from January 2004 to December 2023 using the reported odds ratio (ROR) and information component (IC) methods to assess muscle-related ADR signals.

**Results:**

A total of 18,386 reports of statin myopathy-associated adverse reactions, 348 colchicine myopathy-associated adverse reactions, and 461 muscle-associated adverse reactions due to the combination of the two were collected; the strongest signals of statin myotoxicity events were for necrotizing myositis (ROR 50.47, 95% CL 41.74–61.01; IC 3.70 95% CL 3.25–4.08); the strongest signal for colchicine myotoxicity events was toxic myopathy (ROR 32.50, 95% CL 19.74–53.51; IC 4.97 95% CL 1.89–5.10), and the strongest signal for statins combined with colchicine was toxic myopathy (ROR 159.85, 95% CL 111.60–228.98; IC 7.22 95% CL 3.59–5.9); muscle-related adverse reactions signals were meaningful when the two drugs were combined in the order of colchicine combined with fluvastatin (ROR 187.38, 95% CL 96.68–363.17; IC 6.99 95% CL 1.65–5.68); colchicine combined with simvastatin in 135 cases (ROR 30.08. 95% CL 25.25–35.85; IC 4.80 95% CL 3.96–5.12); and colchicine combined with rosuvastatin (ROR 25.73, 95% CL 20.16–32.83; IC 4.59 95% CL 3.38–4.98) versus colchicine combined with atorvastatin (ROR 25.73, 95% CL 22.33–29.66; IC 4.59 95% CL 3.97–4.91) with almost identical signal intensity, followed by colchicine combined with pravastatin (ROR 13.67, 95% CL 9.17–20.37; IC 3.73 95% CL 1.87–4.47), whereas no signals were generated for lovastatin or pitavastatin.

**Conclusion:**

Similar ADRs can occur when colchicine and statins are used individually or in combination; however, the strength of these reactions may differ. To minimize the risk of drug interactions, statins with less potential interactions, such as lovastatin, pitavastatin, and pravastatin, should be chosen, and myopathy-related indices and symptoms should be closely monitored during use.

## 1 Introduction

Statins have been established as the preferred medication for cholesterol-lowering therapy. Multiple studies have demonstrated that statins can effectively reduce cardiovascular events in patients with atherosclerotic cardiovascular disease (ASCVD) (Cholesterol Treatment Trialists’ CTT Collaboration et al., 2010; LaRosa et al., 2005). By competitively inhibiting hydroxymethlyglutaryl coenzyme A (HMG-CoA) reductase, statins can reduce cholesterol biosynthesis and upregulate cell surface low-density lipoprotein (LDL), which accelerates the catabolism of serum LDL. This leads to a significant reduction in serum total cholesterol (TC), low-density lipoprotein cholesterol (LDL-C), and apolipoprotein B (ApoB) levels, while also mildly lowering serum triglyceride (TG) levels and elevating high-density lipoprotein cholesterol (HDL-C) levels (Slater and MacDonald, 1988). Various types of statins have been introduced for the prevention and treatment of hypercholesterolemia, mixed hyperlipidemia, and cardiovascular diseases. These statins include simvastatin, lovastatin, pravastatin, fluvastatin, atorvastatin, cerivastatin, rosuvastatin, and pitavastatin. Adverse reactions to statins, such as myopathy and rhabdomyolysis, are often a major concern in clinical use due to their impact on muscles (Joy and Hegele, 2009). Among the above statins, cerivastatin was withdrawn from the market in August 2001 because of the risk of death from rhabdomyolysis.

Colchicine is a tricyclic, lipid-soluble alkaloid that is extracted from autumn crocus. It has been extensively studied for its anti-inflammatory properties, owing to its ability to inhibit microtubule polymerization and reduce the levels of several inflammatory molecules, including C-reactive protein (CRP) and interleukin-6 (IL-6), and to resist inflammation (Leung et al., 2015). With two large clinical studies, LoDoCo-2 (Tardif et al., 2019) and COLCOT (Nidorf et al., 2013), suggesting that colchicine can be used to treat coronary heart disease, the U.S. Food and Drug Administration approved the use of low-dose colchicine to reduce the risk of myocardial infarction, stroke, coronary revascularization, and cardiovascular death in June 2023. It can be predicted that the number of patients using both colchicine and statins will increase each year with the increased indications for colchicine-based drugs.

There has been significant clinical interest in the reported myopathy associated with the combination of colchicine and statins. To better understand the characteristics of these adverse drug reactions, this study analyzed the occurrence of myotoxicity due to this combination by FAERS signal mining. This study offers valuable insights into the occurrence of muscle toxicity and can inform clinical decisions regarding the use of these medications together.

## 2 Methods

### 2.1 Data sources

The study data were procured from the FAERS database, which has been publicly available since the first quarter of 2004 and is updated and published quarterly; it is a free and publicly available self-reporting system that, to date, has reported tens of millions of case reports of adverse drug events worldwide. We downloaded all FARES database information from Q1 2004 to Q4 2023. Each section’s quarterly file encompassed seven components, comprising data on patient demographics and administration (DEMO), drug details (DRUG), patient outcomes (OUTC), adverse events (REAC), reporting sources (RPSR), indications for drug administration (INDI), and the start and end dates of therapy for the reported drugs (THER). The FAERS database contains anonymized reported patient information and, therefore, is not subject to ethical approval or informed consent.

### 2.2 Data extraction

The extracted data were entered into the SQL Server 2017 software, and duplicate data were removed from the Error Report, Personal Message Record (DEMO) table according to the “case id” in the Deleted folder provided by the official organization (Fan et al., 2019; Yang et al., 2022). The primary suspected drug was colchicine or statins. In this study, MedEx 1.3.8 software was used to pinpoint the names of specific drugs—both their generic and trade names. To classify adverse drug reactions (ADRs), we employed preferred terms (PTs) from the International Medical Dictionary for Regulatory Activities (MedDRAs) and sorted the data by system organ class (SOC). The data collection centered on ADEs related to myopathy was coded by MedDRA (narrow: 20000002).

### 2.3 Statistical analysis

Excel and SQL Server 2017 software were used for data extraction, organization, and analysis in the present study. The reported cases were statistically analyzed using an Excel sheet for sex, age, occupation of the reporter, region of reporting, outcome of adverse reactions, and number of cases reported per year. This research applied the proportional imbalance method to extract signals utilizing two approaches: the reporting odds ratio (ROR) and the information component (IC). It is worth noting that the proportional imbalance method currently represents the most widely utilized approach for detecting signals associated with ADRs (Zhai et al., 2019). The ROR method calculates the ratio of target ADRs to reported cases of the target drug divided by the ratio of other ADRs to report cases of other drugs. This method is convenient for calculation and has good consistency (Sakaeda et al., 2013). An adverse event was identified when the number of reported cases of the adverse event was ≥3 and the lower 95% CI (ROR_025_) was >1 (Bate and Evans, 2009). The ROR value is used to determine the risk of adverse reactions associated with the target drug, where a higher ROR indicates a greater risk. However, the small number of reported cases can lead to false positives with the ROR method. To reduce this risk, the IC method, which generates an ADR signal when the 95% CI is >0, was used in this study (Khaleel et al., 2022). A larger IC is indicative of a stronger statistical association between the target drug and the occurrence of the target’s ADR.

## 3 Results

### 3.1 Recognition process for adverse drug reactions to colchicine and statins

**FIGURE 1:**
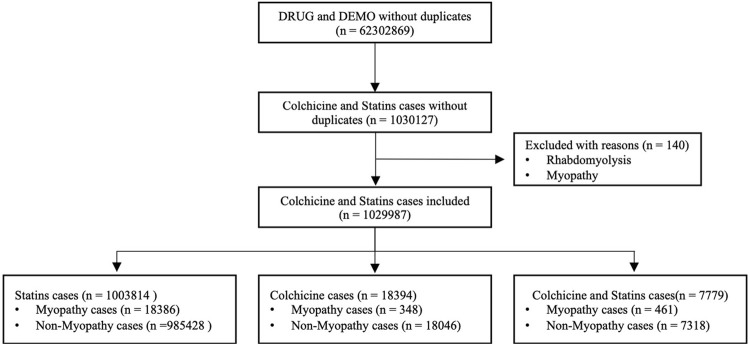
Flow chart of identifying myopathy cases out of colchicine and statins cases from FAERS database.

Positioning the primary suspected drugs as statins and colchicine, a total of 1,030,127 adverse reactions were retrieved. After excluding patients with rhabdomyolysis or myopathy before colchicine and statin use, a final total of 1,029,987 reactions were included. After data extraction, a total of 18,386 reports of statin myopathy-related adverse reactions and 985,428 non-myopathy-related adverse reactions were retrieved from FAERS; 348 reports of colchicine-associated myopathy-related adverse reactions and 18,046 non-myopathy-related adverse reactions were retrieved; and a total of 461 reports of myopathy-related adverse reactions and 7,318 non-myopathy-related adverse reactions were retrieved from the combination of two drugs. The flow chart for identifying myopathy cases with colchicine and statins from the FAERS database is shown in Figure 1.

### 3.2 General characteristics and temporal trends

**FIGURE 2:**
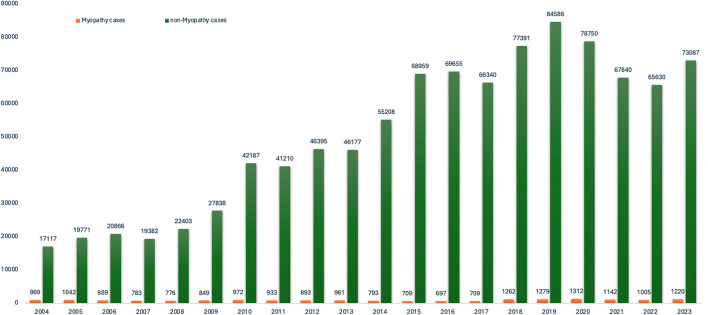
The pertinent annual drug reports on adverse reactions.

As shown in Table 1, in terms of age, 62 patients aged <18 years, 6,364 patients aged 18–64 years, 9,372 patients aged ≥65 years, and 3,397 patients with uncertainty represented the two types of drug myopathy-related adverse reactions groups, with the largest proportion of patients aged >65 years accounting for 48.8%. In terms of sex, males were more common, with a total of 10,772 patients, accounting for 56.10%. The largest proportion of the reported population was professionals with a pharmaceutical background, with 15,979 cases (83.20%), and the countries with the highest number of reports of drug-associated myopathy were Europe, followed by North America and Asia. The most common aspect of adverse outcomes was prolonged hospitalization (51.30%). The details of the no-myopathy patients are also shown in Table 1. The pertinent annual drug reports on adverse reactions are presented in Figure 2. As shown in Figure 2, the number of muscle-related adverse reactions caused by colchicine combined with statins generally increased.

**TABLE 1:** Demographic characteristics of cases.

	Myopathy cases		non-Myopathy cases	
	case /n	proportion/ %	case /n	proportion/ %
Grug group				
Statins	183,86	95.80%	985,428	97.50%
Colchicine	348	1.80%	18,046	1.80%
Colchicine and Statins	461	2.40%	7,318	0.70%
Age group				
<18 years	62	0.30%	2,140	0.20%
18 to 64 years	6,364	33.20%	302,570	29.90%
≥65 years	9,372	48.80%	449,784	44.50%
unknown	3,397	17.70%	256,298	25.40%
Sex				
Female	6,763	35.20%	490,119	48.50%
Male	10,772	56.10%	459,785	45.50%
unknown	1,660	8.60%	60,888	6.00%
Reporter occupation				
Health professionals	15,979	83.20%	525,991	52.00%
non-Health professionals	1,784	9.30%	413,097	40.90%
unknown	1,432	7.50%	71,704	7.10%
Reporter region				
Europe	8,158	42.50%	231,385	22.90%
North America	6,871	35.80%	647,588	64.10%
Asian	1,673	8.70%	42,010	4.20%
Oceania	553	2.90%	9,737	1.00%
South America	72	0.40%	19,512	1.90%
Africa	39	0.20%	3,018	0.30%
unknown	1,829	9.50%	57,542	5.70%
Outcomes				
Death	1,648	8.60%	82,018	8.10%
Life threatening	2,529	13.20%	39,200	3.90%
Hospitalization	9,846	51.30%	303,608	30.00%
Disability	529	2.80%	22,615	2.20%
Congenital anomaly	6	0.00%	572	0.10%
Required intervention	120	0.60%	2,969	0.30%
Other serious events	4,145	21.60%	268,360	26.50%
unknown	372	1.90%	291,450	28.80%

### 3.3 The number of ADRs to colchicine and statins

Among the statins and colchicine used as the first suspected drugs, a total of 18,386 ADRs with a SOC classification of “rhabdomyolysis/myopathy” were screened, of which 6,606 were reported for simvastatin, 6,029 for atorvastatin, 3,467 for rosuvastatin, 569 for pravastatin, 229 for fluvastatin, 226 for lovastatin, 143 for pitavastatin, 31 for cerivastatin, 1,086 for a combined of two statins, and 348 for colchicine. In terms of non-myopathy ADRs, simvastatin 241,514, atorvastatin 422,290, resuvastatin 167,275, pravastatin 83,754, fluvastatin 6,869, lovastatin 24,178, pitavastatin 8,154, cerivastatin 145, the combination of two statins 31,249, and colchicine 18,046 were used. See Table 2 for details.

**TABLE 2:** The number of adverse reactions to colchicine and statins.

	Myopathy cases		non-Myopathy cases	
	case/n	proportion/%	case/n	proportion/%
Drug name				
Simvastatin	6,606	34.40%	241,514	23.90%
Atorvastatin	6,029	31.40%	422,290	41.80%
Rosuvastatin	3,467	18.10%	167,275	16.50%
Pravastatin	569	3.00%	83,754	8.30%
Fluvastatin	229	1.20%	6,869	0.70%
Lovastatin	226	1.20%	24,178	2.40%
Pitavastatin	143	0.70%	8,154	0.80%
Cerivastatin	31	0.20%	145	0.00%
≥2 statins	1,086	5.70%	31,249	3.10%
Colchicine	348	1.80%	18,046	1.80%

### 3.4 Analysis of muscle-related ADR signals to colchicine and statins

**FIGURE 3:**
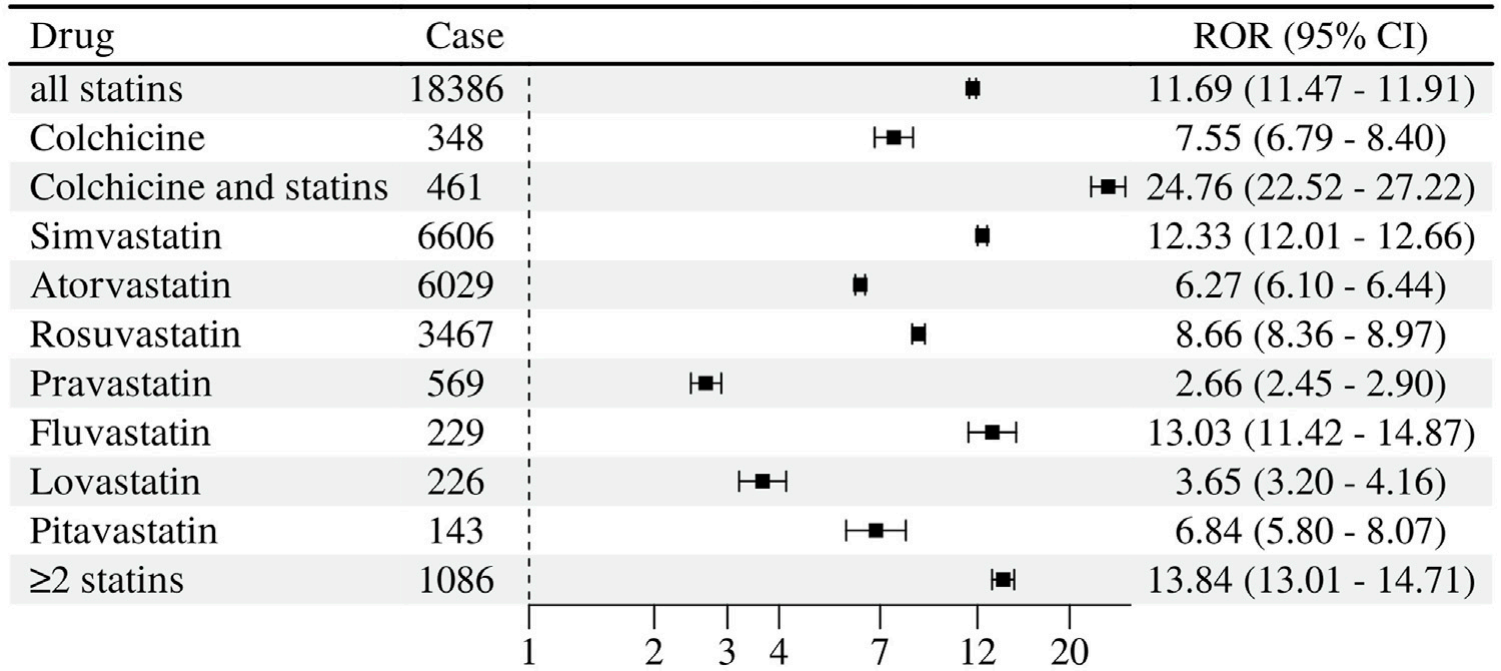
Signal detection of muscle-related adverse reactions of statins and colchicine.

As shown in Figure 3, in terms of generating muscle-related ADR signals, different statins differed in signal strength when used alone. Among them, the strongest was fluvastatin (ROR 13.03, 95% CL 11.42–14.87; IC 3.65 95% CL 3.15–4.02), followed by simvastatin (ROR 12.33, 95% CL 12.01–12.66; IC 3.38 95% CL 3.29–3.46), resuvastatin (ROR 8.66, 95% CL 8.36–8.97; IC 2.99 95% CL 2.87–3.10), pitavastatin (ROR 6.84, 95% CL 5.80–8.70; IC 2.75 95% CL 2.14–3.24), atorvastatin (ROR 6.27, 95% CL 6.10–6.44; IC 2.46 95% CL 2.37–2.55), lovastatin (ROR 3.65, 95% CL 3.20–4.16; IC 1.85 95% CL 1.40–2.27), pravastatin (ROR 2.66, 95% CL 2.45–2.90; IC 1.40 95% CL 1.12–1.67), and a combination of two statins (ROR 13.84, 95% CL 13.01–14.71; IC 3.71 95% CL 3.49–3.90); colchicine also have signals of myopathy toxicity (ROR 7.55, 95% CL 6.79–8.40; IC 2.88 95% CL 2.50–3.21).

### 3.5 Analysis of colchicine and statin PT assays

Further analysis of the muscle-related ADRs associated with the use of statins revealed that the five most common ADRs were necrotizing myositis in 438 cases (ROR 50.47, 95% CL 41.74–61.01; IC 3.70 95% CL 3.25–4.08); myopathy in 3197 cases (ROR 12.60, 95% CL 12.03–13.20; IC 2.91 95% CL 2.77–3.05); rhabdomyolysis 14,716 cases (ROR 11.95, 95% CL 11.70–12.21; IC 2.86 95% CL 2.79–2.92); blood myoglobin was elevated in 528 cases (ROR 10.53, 95% CL 9.43–11.74; IC 2.76 95% CL 2.41–3.08); muscle necrosis 276 cases (ROR 9.40,95% CL 8.10–10.90; IC 2.66 95% CL 2.17–3.09).

The number of muscle adverse events associated with colchicine that generated a signal were as follows:16 cases of toxic myopathy (ROR 32.50, 95% CL 19.74–53.51; IC 4.97 95% CL 1.89–5.10); 128 cases of myopathy 16.82 (ROR 16.81, 95% CL 14.11–20.04; IC 4.04 95% CL 3.29–4.46); 211 cases of rhabdomyolysis (ROR 5.76, 95% CL 5.02–6.60; IC 2.50 95% CL 2.02–2.92); and necrotizing myositis, muscle necrosis, and myoglobinuria, with fewer than three reported cases of urinary myoglobin detection, myoglobinuria, and blood myoglobin detection, which did not qualify for generating the signal conditions.

**FIGURE 4:**
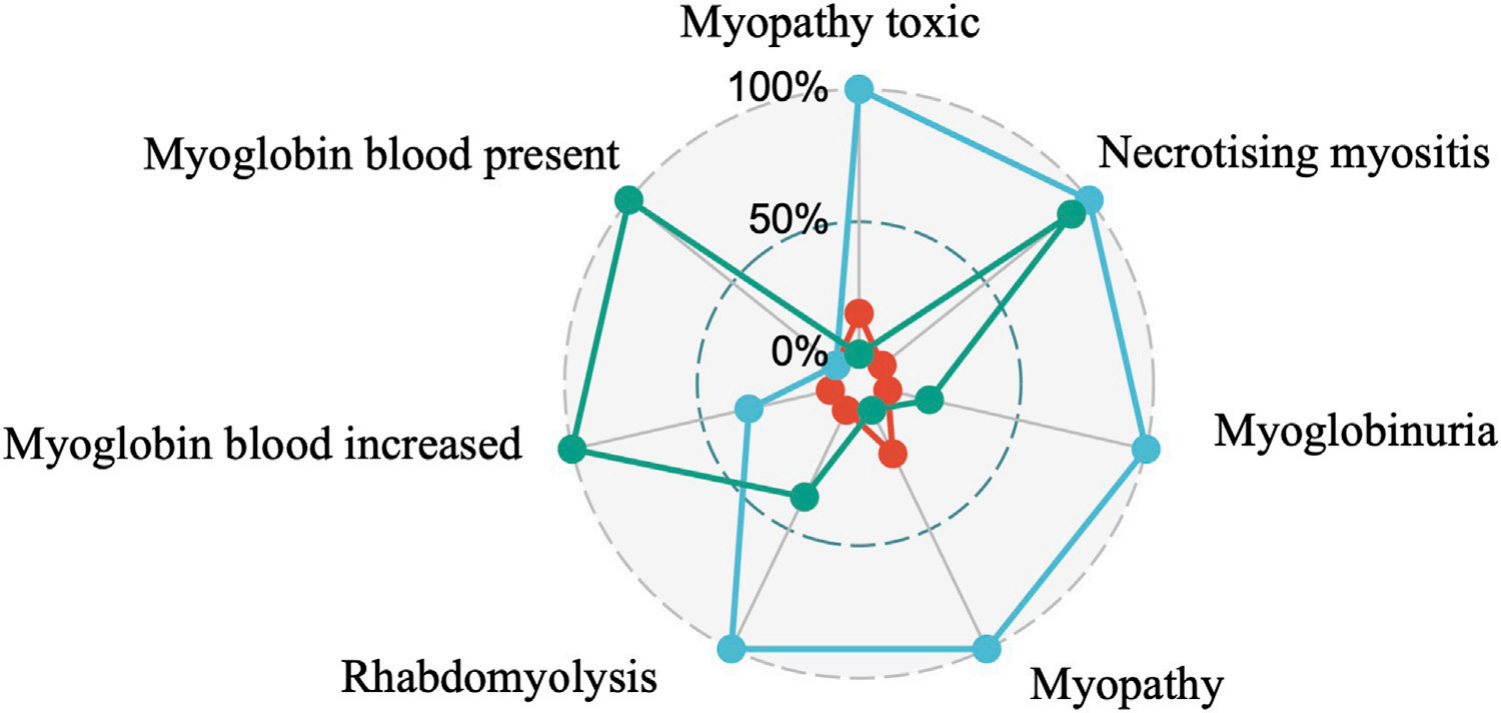
Analysis of the top five myopathy-related adverse events.

About the combination of colchicine and statins, the most frequently detected signal was toxic myopathy in 32 cases (ROR 159.85, 95% CL 111.60–228.98; IC 7.22 95% CL 3.59–5.9), followed by necrotizing myositis in 14 cases (ROR 55.20, 95% CL 32.47–93.85; IC 5.75 95% CL 1.87–5.28), 114 cases of myopathy (ROR 35.65, 95% CL 29.58–42.95; IC 5.11 95% CL 4.13–5.36), and 340 cases of rhabdomyolysis (ROR 22.78, 95% CL 20.42–25.41; IC 4.43 95% CL 3.99–4.71). The number of reported cases of elevated blood myoglobin, urinary myoglobin, myoglobinuria, and blood myoglobin was less than 3. Muscle necrosis was reported in 5 cases (ROR 14.87, 95% CL 6.17–35.85; IC 3.88 95% CL −0.51 to 4.83), myoglobinuria in nine cases (ROR 42.09, 95% CL 21.76–81.43; IC 5.37 95% CL 0.96–5.12) but the lower IC limit of both was less than 0. The above adverse events still need further observation. The corresponding MedDRA codes for PT are shown in the Appendix. Figure 4 shows the analysis of the five most common myopathy-related adverse events associated with colchicine and statin. The PT corresponding to the MedDRA code is shown in Supplementary Table S1.

### 3.6 Analysis of muscle-related ADR signals to colchicine combined with statins

**FIGURE 5:**
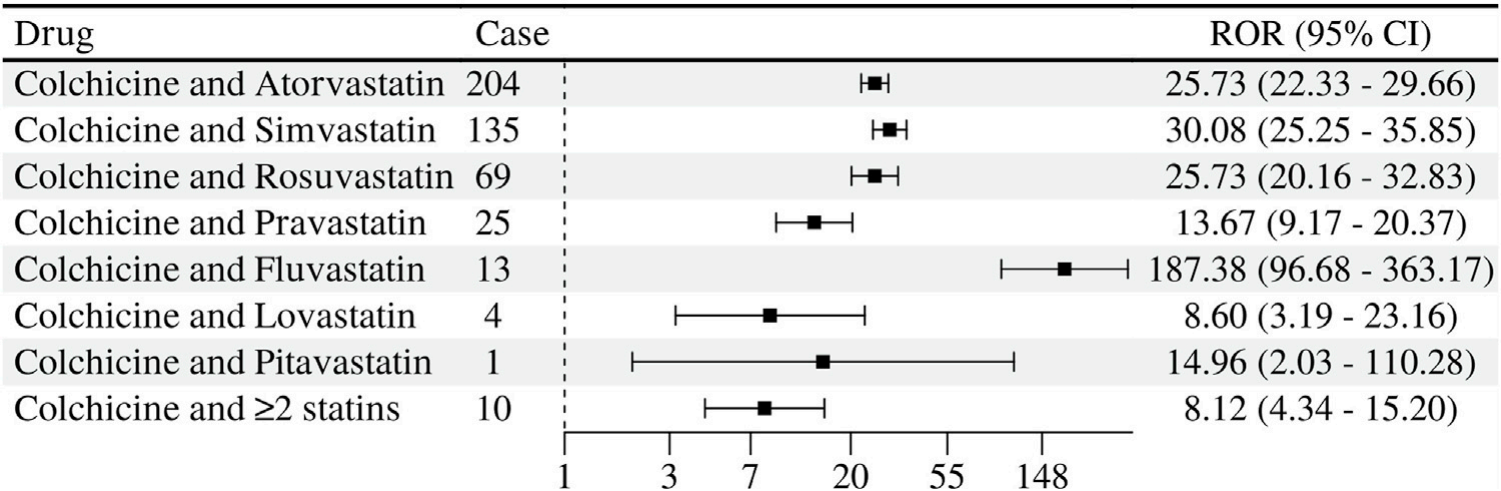
Signal detection of muscle-related adverse reactions of statins and colchicine co-administration.

Different statins used in combination with colchicine had different signals for the detection of myopathic ADRs. The strongest signal strength detected was colchicine combined with Fluvastatin in 13 cases (ROR 187.38, 95% CL 96.68–363.17; IC 6.99 95% CL 1.65–5.68); in second place was colchicine combined with simvastatin in 135 cases (ROR 30.08, 95% CL 25.25–35.85; IC 4.80 95% CL 3.96–5.12); colchicine combined with rosuvastatin (ROR 25.73, 95% CL 20.16–32.83; IC 4.59 95% CL 3.38–4.98) was almost identical to colchicine combined with atorvastatin (ROR 25.73, 95% CL 22.33–29.66; IC 4.59 95% CL 3.97–4.91) signal intensity was almost the same in combination with pravastatin (ROR 13.67, 95% CL 9.17–20.37; IC 3.73 95% CL 1.87–4.47). In contrast, colchicine and lovastatin (ROR 8.60, 95% CL 3.19–23.16; IC 3.08 95% CL −1.19 to 4.71) with colchicine and pitavastatin (ROR 14.96, 95% CL 2.03–110.28; IC 3.85 95% CL −3.87 to 5.67) with IC lower limit of <0 along with pitavastatin had a reported number of only 1, with a reported the number is less than 3. The association of myopathy related ADRs occurring with the combination of drugs still needs to be observed soon. See Figure 5.

## 4 Discussion

Drug-induced muscle adverse reactions refer to the damage caused to muscle tissue and cells by various drugs, encompassing conditions such as myalgia, myopathy, myositis, myonecrosis, and rhabdomyolysis (Rosenson et al., 2014). It typically refers to the emergence of myopathic symptoms in individuals without prior muscle disease after exposure to certain drugs. Both colchicine and statins can cause myopathy, and the myopathy associated with statins and colchicine may present as non-specific muscle pain and weakness. Myopathies most often occur in larger muscles, including the thighs and buttocks. Colchicine and statins are often used together for the treatment of cardiovascular disease, and the current literature suggests that the combination of colchicine and statins may increase the risk of myopathy (Schwier et al., 2022).

Colchicine-induced myopathy is a medical condition that is primarily characterized by the presence of myalgia, muscle weakness, and sometimes rhabdomyolysis. This condition is often characterized by the accumulation of autophagic vacuoles and lysosomal dysfunction, which can be confirmed through muscle biopsy (Slobodnick et al., 2018). At present, the precise way colchicine induces myopathy remains unclear. One potential explanation is that colchicine reduces the formation of microtubules, which are critical for the development of endosomes and autophagosomes. The resultant decrease in microtubules results in the buildup of endosomes and autophagosomes, ultimately leading to myopathy (Uri and Biavis, 1996). Although statins have proven effective and safe, their muscle toxicity remains a significant factor that limits their clinical usage. This toxicity can manifest in various muscle syndromes, including myalgia, myopathy, myositis, and even severe muscle necrosis such as rhabdomyolysis syndrome, for which the incidence may be as low as 0.1% (Grundy, 2002; Wilbur and Makowsky, 2004). Extensive research is currently being conducted to determine the cause of statin-induced myopathy, including genetic studies (Graham et al., 2004). Current theories suggest that myopathy may be caused by an immune-mediated response in which autoantibodies recognize HMG-CoA reductase. These antibodies can have a direct impact on muscle tissues that express HMGCR, leading to symptoms such as myalgia, myopathy, myonecrosis, and significant muscle fiber necrosis, as evidenced by muscle biopsy results (Brunham et al., 2018).

The occurrence of myopathy resulting from the use of both colchicine and statins is currently being investigated and not yet fully understood. It has been suggested that interactions between the drugs may cause increased levels of colchicine or statins in the bloodstream and that this could be due to competition for the same metabolic enzymes and elimination mechanisms (Mammen, 2016). It has been reported that colchicine exhibits moderate inhibition of P-glycoprotein (P-gp) transporter proteins and is also a substrate for CYP3A4 enzymes (Kellick et al., 2014). Statins are typically recognized as substrates for cytochrome P450 (CYP) enzymes, P-gp, and organic anion transporting polypeptides (OATPs) transporters (Slobodnick et al., 2018). While colchicine can inhibit the p-glycoprotein-mediated efflux transporter of statins and lead to myopathy, it is also a substrate of CYP3A4 enzymes, as are statins. This can cause competition between the two drugs and result in higher serum concentrations of both, thereby increasing the risk of side effects (Balasubramanian and Maideen, 2021). However, colchicine does not seem to interact with the OATP drug transporter. It is important to note that the combination of these drugs causing myopathy can be multifactorial and not solely due to drug interactions. Other factors, such as advanced age and hepatic or renal insufficiency, should also be considered (Bogman et al., 2001).

Although some statins rely on CYP3A4 metabolism to varying degrees, not all of these effects are eliminated through this process. Simvastatin, lovastatin, and atorvastatin undergo significant CYP3A4 metabolism, whereas pitavastatin, fluvastatin, and rosuvastatin are weak substrates of the enzyme. Pravastatin is the only statin that is not metabolized by CYP450. Additionally, atorvastatin, lovastatin, pitavastatin, and simvastatin are considered P-gp substrates and inhibitors, while rosuvastatin is not eliminated via P-gp (Uri and Biavis, 1996).

The findings of this study indicate that the co-administration of colchicine with various statins resulted in the emergence of myopathic signals, with varying degrees of severity. Specifically, it is observed that the order of myopathy induction from strongest to weakest was fluvastatin, simvastatin, and a comparable incidence of myopathy was noted for rosuvastatin and atorvastatin, followed by pravastatin. Notably, no myopathy-related signals were detected with lovastatin and pitavastatin. These results provide valuable insights regarding the potential risks associated with the use of colchicine in combination with different statins and could help in the development of strategies to mitigate these risks. In the quest for clinical reports on the occurrence of myopathy with the combination of colchicine and statin, a total of 16 cases of simvastatin (Hsu et al., 2002; Baker et al., 2004; Justiniano et al., 2007; Sahin et al., 2008; Oh et al., 2012; Boonmuang et al., 2013; Frydrychowicz et al., 2017; Kwon et al., 2017), 13 cases of atorvastatin (Phanish et al., 2003; Atasoyu et al., 2005; Tufan et al., 2006; Sahin et al., 2008; Erlandson and Castillo-Mancilla, 2010; Boonmuang et al., 2013; Patel et al., 2016; Gupta et al., 2019), 3 cases of rosuvastatin (Erlandson and Castillo-Mancilla, 2010; Sarullo et al., 2010), 2 cases of fluvastatin (Atasoyu et al., 2005; Sarullo et al., 2010), 2 cases of pravastatin (Barilla et al., 2004; Torgovnick et al., 2006), and 1 case of lovastatin (Grundy et al., 2019) were examined. Our study found a stronger fluvastatin signal, but it has been suggested that using fluvastatin alone may pose the lowest risk of myotoxicity. This is due to its lower metabolism by CYP3A4, resulting in weaker drug-drug interactions. Additionally, the extended-release tablet dosage form leads to lower systemic exposures (Barilla et al., 2004), whereas with simvastatin, either in existing clinical reports or in the analysis of our study, the number of reported cases and signal strength were higher, so it is recommended to be avoided in clinical use. The signal strengths of atorvastatin and rosuvastatin demonstrate similar efficacy as cholesterol-lowering agents. However, when combined with colchicine, atorvastatin has been associated with a higher incidence of myopathy. This could be attributed to the fact that atorvastatin is predominantly metabolized by CYP3A4. Lovastatin and pitavastatin, which do not produce muscle-related signals and have been less reported, appear to produce fewer muscle-related toxicities in combination with colchicine (Alayli et al., 2005; Francis et al., 2008; Bouquié et al., 2011).

Among muscle-related adverse events, colchicine-related adverse events were toxic myopathy, myopathy, and rhabdomyolysis in order of signal intensity; statins were necrotizing myositis, myopathy, and rhabdomyolysis in that order. The signal order of adverse event detection for the combination of the two drugs was toxic myopathy, necrotizing myositis, myopathy, and rhabdomyolysis. Colchicine, statins, and muscle-related adverse events of colchicine combined with statins lacked specificity. Currently there are studies showing that both drugs have muscle-related side effects, and it is difficult to distinguish between colchicine-induced or statin or other causes of muscle toxicity in actual treatment, so muscle toxicity-related indicators or clinical manifestations should be closely monitored in the period of using both drugs.

When both colchicine and statins are required for a patient’s treatment, certain studies suggest that it may be reasonable to combine colchicine with rosuvastatin, fluvastatin, lovastatin, pitavastatin, or pravastatin (Grundy et al., 2019). However, it is important to closely monitor for muscle-related signs and symptoms to detect any potential synergistic muscle-related toxicity. In cases where atorvastatin, simvastatin, and lovastatin are taken alongside colchicine, it may be necessary to reduce the dosage due to drug interactions. Additionally, if statins are used alongside colchicine for patients with renal insufficiency, it is important to adjust the colchicine dosage accordingly. It is also important to note the presence of other influencing factors for the development of myopathy in patients, such as Advanced age (≥75 years), History of creatine kinase elevation, Vitamin D deficiency, Hepatic impairment, Hypothyroidism, Frailty/low body mass index. For patients receiving concurrent drug therapy, it is imperative to consider the presence of other P-gp and CYP3A4 inhibitors, such as Diltiazem, fluconazole, Azithromycin, carvedilol, verapamil, etc., as they have the potential to impact statin and colchicine concentrations thereby increasing the risk of myopathy (Schwier et al., 2022). Additionally, vigilance should be exercised regarding the co-administration of drugs with myotoxicity properties like Proton pump inhibitors (PPIs) (Capogrosso Sansone et al., 2017).

## 5 Limitations

Notably, the FAERS database operates as a spontaneous reporting system, which presents certain limitations in terms of the accuracy and completeness of the reported data. As such, it is possible for errors, duplications, and omissions to occur in the data, and for subjective descriptive bias to impact the quality of the information. These limitations must be taken into consideration when analyzing the data and drawing conclusions from it (Sakaeda et al., 2013). Although it is currently believed that there may be an underestimation of the occurrence of adverse events in the spontaneous reporting systems (Figueiras et al., 2006), all the results of signal detection can only illustrate the statistical correlation, and further high-quality clinical studies are still needed to confirm the existence of an exact association. The data in this study only analyzed the signal strength of muscle-related adverse events that may occur when colchicine is combined with statins, but in the analysis process, due to the existence of patient dosage, duration of treatment, combined drugs, and other information is partially missing in the dosage, duration of treatment, combined drugs and other aspects of the analysis could not be carried out in-depth, and this part of the impact on the signal relationship needs to be further verified.

## 6 Conclusion

In this study, real-world signal mining and analysis of muscle-related adverse events caused by colchicine in combination with statins were performed using the proportional imbalance method. The results of the study revealed that the muscle-related adverse events of colchicine in combination with statins lacked specificity, and the strongest signal was toxic myositis, consistent with colchicine; when colchicine is used in combination with statins, it is suggested to prioritize lovastatin, pitavastatin, and pravastatin, and to pay attention to the indicators of myopathy related to the combination of drugs and clinical symptoms.

## Data Availability

The original contributions presented in the study are included in the article/Supplementary Material, further inquiries can be directed to the corresponding author.
