# Supplementary material for: Muscular toxicity of colchicine combined with statins: a real-world study based on the FDA adverse event reporting system database from 2004–2023
Source: Front Pharmacol. 2024 Jul 26;15:1418498. doi: 10.3389/fphar.2024.1418498 (PMC11310597; doi:10.3389/fphar.2024.1418498)
Supplement: Supplementary file 1 [file Table1.pdf]

Supplementary Material

Muscular toxicity of colchicine combined with statins: a real-world study based on the FDA adverse event reporting system database from 2004-2023

Ying Liu, Chunyan Wei, Yanling Yuan, Dan Zou, Bin Wu*

*** Correspondence: Bin Wu binw83@scu.edu.cn**

Supplementary Table 1. The Number of Adverse Reactions to Colchicine and Statins

aPT: prefer term.

NO.	PT [a]code	PT [a]name
1	10028320	Muscle necrosis
2	10028625	Myoglobin blood increased
3	10028629	Myoglobinuria
4	10028631	Myoglobin urine present
5	10028641	Myopathy
6	10028648	Myopathy toxic
7	10039020	Rhabdomyolysis
8	10058735	Myoglobinaemia
9	10059888	Myoglobin blood present
10	10074769	Necrotising myositis
11	10081524	Thyrotoxic myopathy
12	10086278	Muscle infarction

aPT: prefer term.
